# Meta-Analysis of Pharmacological, Nutraceutical and Phytopharmaceutical Interventions for the Treatment of Cancer Related Fatigue

**DOI:** 10.3390/cancers15010091

**Published:** 2022-12-23

**Authors:** Sriram Yennurajalingam, Zhanni Lu, Aline Rozman De Moraes, Nhu Nhu Tull, Michal J. Kubiak, Yimin Geng, Clark R. Andersen, Eduardo Bruera

**Affiliations:** 1 Department of Palliative Care Rehabilitation and Integrative Medicine, University of Texas MD Anderson Cancer Center, Houston, TX 77030, USA; 2 Research Medical Library, University of Texas MD Anderson Cancer Center, Houston, TX 77030, USA; 3 Department of Biostatistics, University of Texas MD Anderson Cancer Center, Houston, TX 77030, USA

**Keywords:** cancer, fatigue, metanalysis, pharmacological, nutraceuticals, phytopharmaceuticals, interventions

## Abstract

**Simple Summary:**

In our study we found that the overall meta-analysis of all cancer related fatigue (CRF) treatment studies showed significant reduction of CRF. The meta-analysis did not show significant reduction of CRF with treatment ginseng, guarana, megestrol, mistletoe, psychostimulants, selective serotonin reuptake inhibitors/antidepressants. Metanalysis of Corticosteroids studies showed significant reduction in CRF. Further studies are needed.

**Abstract:**

**Purpose:** In this study we aimed to estimate the effectiveness of pharmacological, nutraceutical, and phytopharmaceutical treatments on CRF. **Methods:** Ovid MEDLINE, Ovid Embase, Ovid Psych info, CINHAHL and Cochrane Library databases were searched up to 30 September 2021. Randomized controlled trials of pharmacological, nutraceutical and phytopharmaceutical interventions for treatment of CRF for at least one week duration and have used valid tool to assess severity of CRF as a primary or secondary outcome were considered. **Results**: 32 eligible studies (4896 patients) were reviewed. For the overall meta-analysis, the random effect models yielded the treatment effect (95% CI) of −0.29 (−0.48,−0.09), *p* < 0.001. The meta-analysis did not show significant reduction of CRF with treatment with ginseng (n = 6), guarana (n = 3), megestrol (n = 2), mistletoe (n = 3), psychostimulants (n = 14), SSRI/antidepressants (n = 2). Corticosteroids (n = 2) showed significant reduction in CRF with treatment effects of 0.94 (−1.21, −0.67), *p* <0.0001, respectively. **Conclusions**: In this study, overall meta-analysis of all studies demonstrates significant reduction of CRF using Pharmacological, Nutraceutical and Phytopharmaceutical interventions with a pooled standardized treatment effect of −0.29. Metanalysis of Corticosteroids studies showed significant reduction in CRF. Further studies are needed.

## 1. Introduction

Cancer-related fatigue (CRF) is one of the most common and distressing symptoms associated with cancer and its treatment [1,2,3,4,5]. The frequency of CRF in cancer patients varies from 60% to 90% [1,2,3,4,5,6]. CRF negatively affects quality of life (QOL), interferes with daily activity, has potentially devastating social and economic consequences, affects the patient’s ability to receive cancer therapy, and may potentially negatively impact public health [2,6,7,8,9]. The National Comprehensive Cancer Network defines CRF as a “distressing, persistent, subjective sense of physical, emotional, and/or cognitive tiredness or exhaustion related to cancer or cancer treatment that is not proportional to activity and that interferes with usual functioning” [2].

CRF is pervasive in all cancer patients and survivors but more severe in advanced cancer patients and immediately after cancer treatments [10]. It also can be very debilitating in at risk patients for example in patients with psychological or cachexia related comorbidities [11,12]. While non-pharmacological treatments for CRF have the best evidence, most of these interventions are not feasible due to poor adherence due to progressive disease or associated symptoms such as drowsiness. Additionally, medication seems to be sometimes preferable among certain patients for the treatment of CRF due to ease of use, and this treatment may offer best expectation of improvement. Various pharmacological and nutraceuticals medications have been used for the treatment of CRF in both clinical trials and practice. Some of these nutraceuticals are often self-prescribed by the patients. The most investigated medications among them include psychostimulants, ginseng, corticosteroids, megestrol, mistletoe, guarana, antidepressants specifically selective serotonin receptor reuptake inhibitors (SSRI’s). Other agents investigated include Hemopoietin agents [13], Donepezil [14], Testosterone [15,16], Omega 3 polyunsaturated fatty acids [17,18,19], L-carnitine [20,21,22], Astragalus membranaceus [23], Ashwagandha [24], Thyrotropin releasing hormone [25], Microbiome [26], Coenzyme Q10 [27], Micro and macronutrients supplementation such as protein rich diet, vitamin or mineral supplementation [28], and others [29,30,31,32].

CRF is a complex subjective symptom due its multidimensional nature, and pathophysiological mechanisms may vary based on the stage of disease, treatment, and presence of comorbidities [1,2,3,4,5]. Majority of these factors impact the brain where CRF is perceived [33]. Different known factors result in the multidimensional manifestation of CRF including: Among them the most common factors include (a) central nervous system [CNS] factors including disturbance in cognition, sleep/wakefulness and mood (anxiety and depression), dysregulation of hypothalamic pituitary axis, autonomic nervous system, and circadian rhythm [33]; (b) dysregulation of inflammatory cytokines including IL-6, TNF-alpha, IL-1 and its receptor); and (c) reduced function (physical activity, deconditioning). Briefly, the molecular mechanisms of the commonly investigated agents used to treat CRF is unclear but target one these mechanisms.

Methylphenidate is a CNS stimulant. The mechanism by which it improves CRF may be due to its action by blockage of presynaptic dopamine and norepinephrine reuptake [34], reticular activating system (arousal), and its effects on mood, can impact the physical, cognitive, functional, and psychological contributors to CRF [35].

Like Methylphenidate, Modafinil and its Armodafinil may improve CRF by non-noradrenergic, dopamine-dependent adrenergic signaling in the wake-promoting mechanism [36]. Megestrol is a progestin is commonly used advanced cancer patients to improve anorexia. The mechanism of action it may improve CRF is by its effect on the hypothalamic-pituitary-gonadal axis [37]. One of the hypotheses about megestrol acetate’s mechanism of action is that it has inhibition of inflammatory cytokines such as tumor necrosis factor, IL-1, and IL-6 [37,38].

SSRI/Antidepressants: Antidepressants with selective serotonin reuptake inhibitors (SSRI) activity with and without norepinephrine (NE) and dopamine neurotransmitter systems have been used for the treatment of CRF. The exact molecular mechanisms are not clear but the likely related to the effects of SSRI’s on depression, as CRF and depression share some of the causative mechanisms such as dysregulations in inflammatory pathways, dysfunction of the hypothalamic pituitary axis, as well as psychostimulant like action [39,40].

Corticosteroids have direct central nervous system effects, including modulation of the hypothalamic pituitary axis and inflammatory cytokines such as IL-1, IL-6, TNF-alpha and their receptors [41], and effects on mood, which all cause the drug to impact the physical, cognitive, functional, and psychological contributors of CRF [42].

Ginseng is derived from processed plant root of ginseng species (Panax Ginseng, *Panax quinquefolius* L., *Eleutherococcus senticosus* (Rupr. & Maxim) [43]. Ginsenosides and saponins are the important pharmacological ingredients [44]. Possible mechanisms proposed by which ginseng may improves CRF is by its action on (a) CNS, including cognition/memory, sleep disturbance, anxiety/depression, (b) by Neuroprotection via potentiation of nerve growth factor activity [45,46], (c) inhibition of the activity of *N*-methyl-d-aspartate(NMDA) receptor activity [47], (d) inhibition of excitatotoxicity and calcium overflux into neurons, increase dopamine and norepinephrine in the cerebral cortex [48] and modulate the activity of presynaptic and postsynaptic receptors, and (e) modulation of inflammatory cytokines [49,50].

Guarana (*Paullinia cupana*) extract has been used to treat CRF, and it has high concentration of caffeine (2.5 and 5.8% of the fruit’s dry weight) [51,52], theobromine, theophylline (both below 0.3%) and flavonoids (proanthocyanidins, catechins, and epicatechines) [53,54]. The exact mechanism of improvement of CRF is unclear but may be due these compounds [55].

Mistletoe (*Viscum album* L.) total plant extracts have been used for the treatment of CRF [56]. Possible role in improvement in CRF may be related to its immunomodulatory effect [57].

However, there are very limited recent meta-analysis conducted using rigorous methods including all pharmacological and nutraceuticals medications used for the treatment of CRF despite several full systematic reviews [2,33,58,59,60,61,62,63]. Due to which the current guidelines from various professional organizations such as American Society of Clinical Oncology (ASCO), NCCN, and European Society of Medical Oncology (ESMO) provide limited indications for the use of pharmacological or nutraceuticals [64,65]. Hence, to date there is no FDA approved pharmaceutical or nutraceutical treatment for cancer related fatigue. Therefore, in this study we aimed to conduct a metanalysis of pharmacological, nutraceutical, and phytopharmaceutical treatments on CRF to estimate their effectiveness to treat CRF.

## 2. Methods

This study was approved by the institutional review board (IRB) of the University of Texas, MD Anderson Cancer Center, Houston Texas. Our meta-analysis has been registered with PRISMA Transparent reporting of systematic reviews and meta-analyses (http://www.prismastatement.org/Protocols/Registration accessed on 1 November 2022). The project ID in the PRISMA/PROSPERO website is CRD42021203093.

We reviewed pharmacological, nutraceutical, and phytopharmaceutical Intervention studies in adult cancer patients on CRF receiving treatment or during post-treatment. We had five reviewers (SY, ARM, ZL, NN, and MK), to assess the inclusion and exclusion of the studies via reviewing the titles, abstracts, and full texts. The reviewers were randomly assigned to two groups and each group received half of the papers identified by the librarian (GY). Individually, each member assessed the inclusion and exclusion criteria and then cross checked the results. In case of conflicts, the study principal investigator (SY) made the final decision whether include or not a given study.

## 3. Study Selection

To be eligible to be included in the study, the articles had to meet the following eligibility criteria: (a) English language publications, (b) available in Ovid MEDLINE, Ovid Embase, Ovid Psych info, CINHAHL and Cochrane Library databases, (c) pharmacological, nutraceutical, or phytopharmaceutical interventions in human subjects with cancer, (d) Use randomized clinical trial phase II or III design, (e) Timeline—from 1 January 1967 to 30 September 2021, (f) Use a valid and acceptable fatigue outcome measures [66] such as Functional Assessment of Chronic Illness Therapy–Fatigue (FACIT-F), Brief Fatigue Inventory (BFI), Piper Fatigue Scale (PFS), Fatigue Symptom Inventory (FSI), Multidimensional Fatigue Symptom Inventory (MFSI), Multidimensional Assessment of Fatigue (MAF), European Organization for the Research and Treatment of Cancer Quality of Life Questionnaire (EORTC-QLQ-30) fatigue, The MD Anderson Symptom Inventory(MDASI), that assess both presence of fatigue and severity of fatigue, (g) cancer related fatigue should be a primary or secondary outcome, (h) The duration of the selected intervention should be at least 1 week, (i) The selected intervention should be compared to a placebo or another intervention or standard care. The articles were excluded if there was: (a) assessment of CRF using treatment toxicity only using the Common Terminology Criteria for Adverse Events (CTCAE) or equivalent, (b) combinations treatments, (e.g., combination treatment of Coenzyme-Q, N acetyl Carnitine), (c) hemopoietin growth factors, (d) less than 30 patients enrolled in the study.

Based on the above strategies, we have included 32 eligible peer-reviewed manuscripts (4896 total patients) that assessed the effects of pharmacological, nutraceutical, and phytopharmaceutical interventions on cancer induced fatigue. These selected peer-reviewed manuscripts involved the interventions of psychostimulants (Methylphenidate, Dexamphetamine, Modafinil and armodafinil); corticosteroids (Dexamethasone and Methylprednisolone); selective serotonin reuptake inhibitors (SSRI’s); Ginseng; Guarana; Melatonin; Minocycline; Mistletoe; and Megestrol, and they were assessed using the meta-analysis approach.

## 4. Collection and Analysis

### 4.1. Search Terms Used

- Fatigue And Cancer (Primary/Broad)
- “cancer” “neoplasm”, “tumor”, “oncology”, “fatigue”, “tiredness”, “weary”, “weariness”, “exhaustion”, “exhausted”, “lackluster”, “asthenia”, “lassitude”, “lack of energy”, “drug therapy”, “diet therapy”, “central nervous system stimulants”, ”methylphenidate”, “dextroamphetamine”, “dexmethylphenidate”, “psychostimulants”, “psychotropic”, “modafinil”, “armodafinil”, “pemoline”, “donepezil”, “amantadine”, “etanercept”, “antidepressive agents”, “serotonin uptake inhibitors”, “sertraline”, “paroxetine”, “fluoxetine“, “acetylsalicylic acid”, “aspirin”, “adrenal cortex hormones”, “glucocorticoids”, “corticosteroids”, “steroids”, “dexamethasone”, “methylprednisolone”, “progestins”, “progestational steroids”, “testosterone”, “thyrotropin-releasing hormone”, “erythropoietin”, “darbepoetin”, “adenosine triphosphate”, “thyroliberin”, “fish oils”, “docosahexaenoic acids”, “vitamin D”, “carnitine”, “levocarnitine”, “anticytokine”, “antineoplastic agents”, “medicinal plant”, “herbal medicine”, “phytotherapy”, “mistletoe”, “ginseng”, “paullinia”, “astragalus” and “placebo” etc.

Subject heading searches were explored to include narrower terms in the Medical Subject Headings (MeSH) or EMTREE (subject headings unique to Embase) hierarchy as needed. The search terms were combined by “or” if they represented the similar concept, and by “and” if they represented different concepts. Searches were restricted to randomized clinical trials of phase II or Phase III design in adult cancer patients. Conference abstracts, systematic reviews and case reports were excluded.

We searched the abstracts of the identified articles from the databases for inclusion in the review. The primary author and the medical librarian independently carried out a study selection to determine that the articles meet the inclusion criteria. Any disagreement about a particular study was resolved by discussion.

### 4.2. Data Extraction

We extracted data from each included study into the following areas of focus: (I) General information: article identification (author, year), full citation, geographic location, setting (e.g., hospital-based, clinic-based, community-based, referral criteria/process, other), declared conflict of interest, and source(s) of funding. (II) Study characteristics including design (Randomized controlled trials (RCT’s), clinical trial, survey, other), duration of study, number of centers, sample size, follow-up assessments, primary outcome (definition, instrument used, scoring), and secondary outcomes (definition, instrument used, scoring) (III) Patient Population details included in the study such as inclusion criteria, exclusion criteria, baseline characteristics (targeted to the specific topic—e.g., mean age, gender (male/female %), description of the therapy (time, intensity, frequency, etc.), and description of the comparator (intensity, time, frequency). (IV) Quality appraisal: Physiotherapy Evidence Database (PEDro) scoring systems was used to evaluate the quality of the articles [67]. PEDro scoring is a checklist on 10 scored yes or no questions. It evaluates internal validity and statistical information. At least two blinded evaluators checked independently each include paper to score it and an average result was calculated. Any major discrepancies were evaluated and discussed with principal investigator. The Cochrane risk assessment tool [68] was used to assess the methodological quality of each study including the risk of selection, performance, detection, attrition, and reporting biases, Each reviewer scored the Cochrane assessment tool individually analyzing the study method of randomization, allocation concealment, blinding of patients, investigator and assessor, incomplete outcome data (participation rate, sampling procedure/sample size calculation, analysis, completion per study design, handling of missing outcome data), selective outcome reporting, and the possibility of other biases. (V) Outcomes: Continuous (fatigue scales such as FACIT-F, BFI, etc.), Group mean (final value or mean change from baseline), Group standard deviation (can be calculated from SE, T-test, *p* values), and total number of patients per group were assessed.

### 4.3. Statistical Analysis

**Figure 1:**
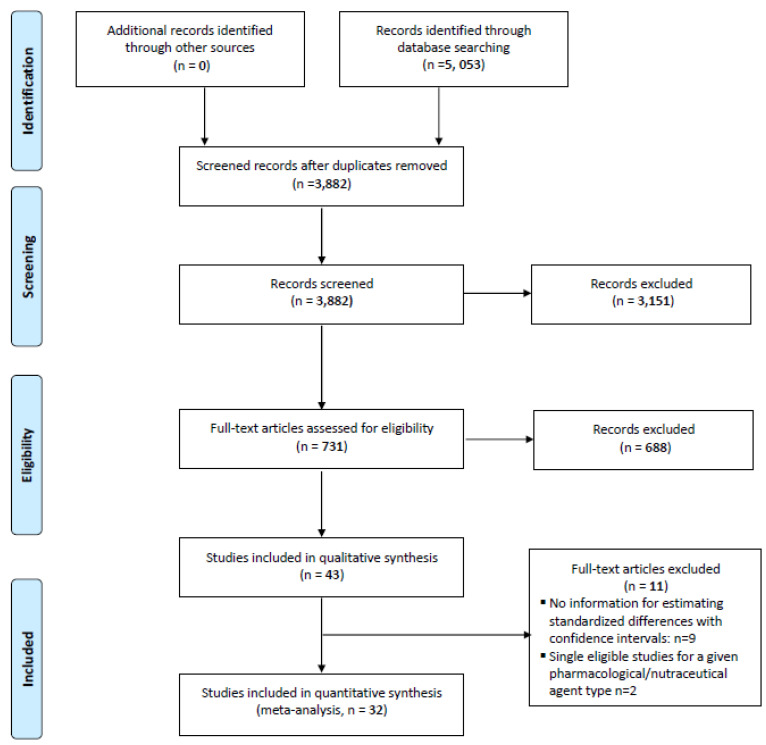
PRISMA FLOW DIAGRAM.

Results were reviewed from 43 studies which assessed the effect of drug treatment on fatigue using a variety of scales. Drug treatments included ginseng, guarana, megestrol, mistletoe, psychostimulants, SSRI/antidepressants, and steroids. For studies which reported results on multiple scales, single scale results were selected, prioritized in order of FACIT-F, then BFI, followed by other scales. To allow meta-analysis of the pooled results from these different scales, reported score differences were converted to standardized mean difference (Z-scores), such that an increase in score corresponds to increase in fatigue, with 95% confidence intervals. Nine studies were excluded because they did not have sufficient information to estimate a standardized difference with confidence intervals, which left 32 studies for the meta-analysis (Figure 1). We excluded the metanalysis of two pharmacological or nutraceutical agents (melatonin and minocycline) as there was only one study for each of these agents which were eligible as per the eligibility criteria of the study [69,70]. These studies were grouped by treatment sub-group analyses. Meta-analyses, both fixed-effect and mixed effect, were performed for each of the treatments as well as overall, using the inverse variance method, with the DerSimonian-Laird estimator for tau^2 and the Jackson method for confidence intervals of tau^2. Cochrane’s Q and I^2 were used to assess heterogeneity among studies. Given the heterogeneous nature of drug treatments and the design and conduct of these studies, the random effects model estimates of effect size, confidence interval, and *p*-values were prioritized over those of the fixed effects models. Therefore, in the results we reported only the random effects model estimates of treatment effects. Corresponding forest and funnel plots were produced. All statistical analyses were performed using R Core Team (2020) [71], with meta-analyses performed using the “meta” package [72].

## 5. Results

**Figure 2:**
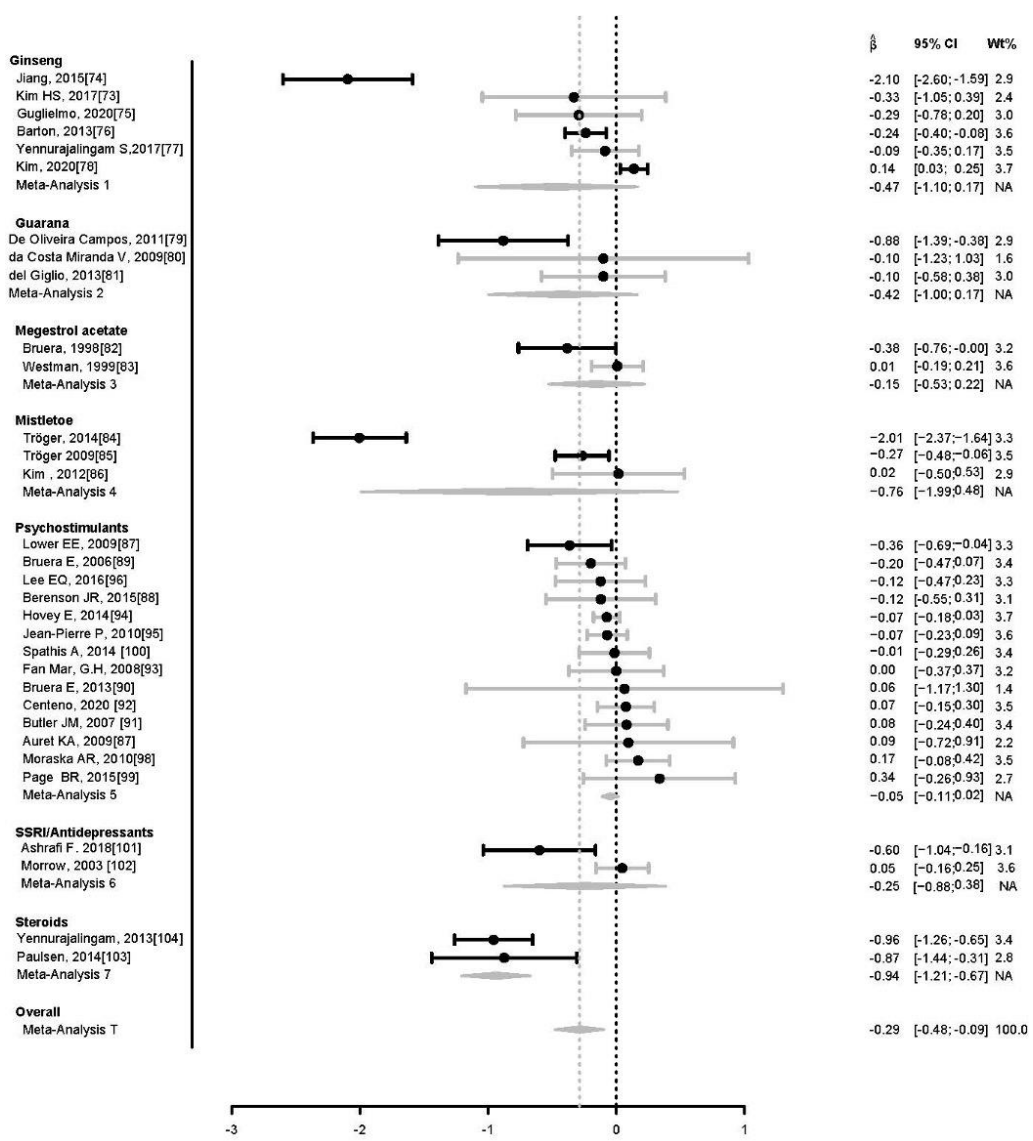
Meta-analyses of pharmacological, nutraceutical, and phytopharmaceutical treatments on Cancer related Fatigue using a forest plot.

**Figure 3:**
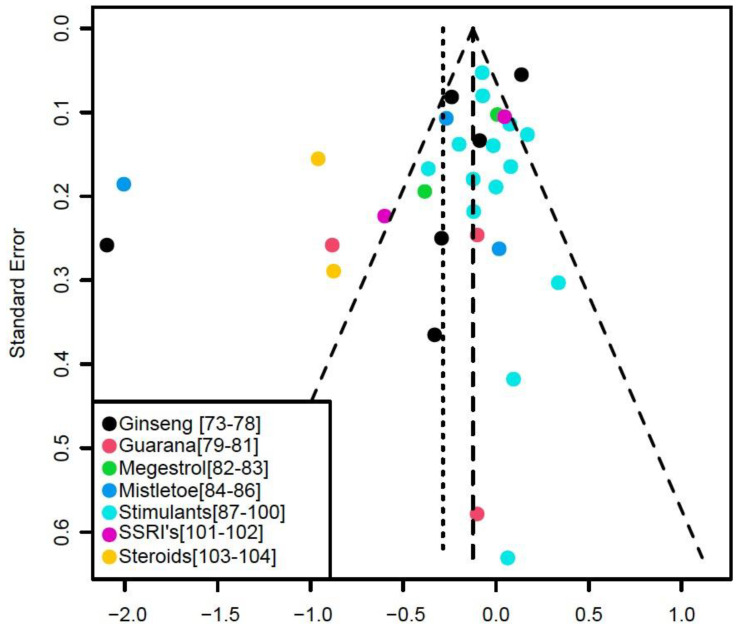
Funnel plot illustrating a scatterplot of study standard errors with relation to treatment effect.

**Figure 4:**
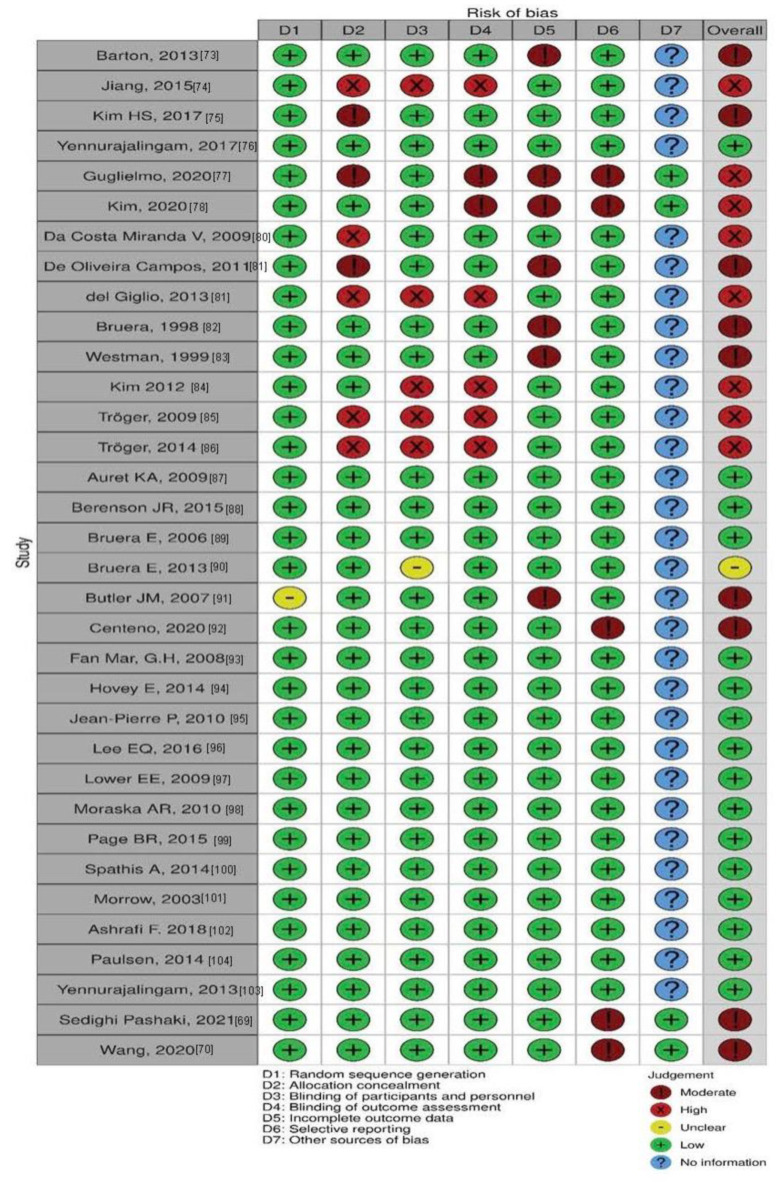
Cochrane risk of bias assessment using traffic-light plot.

**Figure 5:**
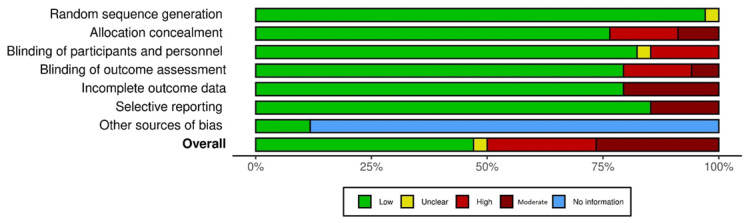
Cochrane risk of bias assessment summary plot.

Among the 32 studies (4896 patients) reviewed (Figure 1, Table 1), 10 studies reported significantly lower fatigue due to treatment. Results of the meta-analyses on the effect of treatments on fatigue are illustrated in the forest plot (Figure 2). Fatigue scales were transformed to standardized mean difference (Z-scores), such that an increase in score corresponds to increase in fatigue. The funnel plot (Figure 3) shows that most treatment effects are clustered near zero and with some evidence of bias towards smaller effect sizes and some heterogeneity. Table 2 shows the Risk Bias for studies included in the Meta-Analysis. Figure 4 and Figure 5 show the Cochrane risk of bias assessment using traffic-light plot, and Cochrane risk of bias assessment summary plot, respectively.

For the overall meta-analysis, the random effect models yielded the estimates of treatment effect and 95% confidence interval of −0.29 (−0.48, −0.09), *p* < 0.001. Cochrane’s Q supports heterogeneity among studies (Q = 305.8 with 33 degrees of freedom, *p* < 0.0001), as did I^2, estimated as 89% (86%, 92%). Tau^2 was estimated as 0.27 (0.16, 0.50), suggesting a low variance of the estimated treatment effect size.

The ginseng meta-analysis of 6 studies [73,74,75,76,77,78], yielded random effects model treatment effects of −0.47 (−1.10, 0.17), *p* = 0.15. Cochrane’s Q supports heterogeneity among studies (Q = 81.3 with 5 degrees of freedom, *p* < 0.0001), as did I^2, estimated as 94% (89%, 97%). Tau^2 was estimated as 0.59 (0.20, 3.87), suggesting a low variance of the estimated treatment effect size.

The guarana meta-analysis of 3 studies [79,80,81] yielded random effects model treatment effects of −0.42 (−1.00, 0.17), *p* = 0.16. Cochrane’s does not support heterogeneity among studies (Q = 5.17 with 2 degrees of freedom, *p* = 0.08), though I^2 does suggest some heterogeneity, estimated as 61% (0%, 89%). Tau^2 was estimated as 0.15 (0.0, 7.9), suggesting a low variance of the estimated treatment effect size.

The megestrol meta-analysis of 2 studies [82,83] yielded random effects model treatment effects of −0.15 (−0.53, 0.22), *p*= 0.43. Cochrane’s Q does not support heterogeneity among studies (Q = 3.17 with 1 degrees of freedom, *p* = 0.08), nor does I^2, estimated as 68% (0%, 93%). Tau^2 was estimated as 0.05, suggesting a low variance of the estimated treatment effect size.

The mistletoe meta-analysis of 3 studies [84,85,86] yielded random effects model treatment effects of −0.76 (−2.00, 0.48), *p* = 0.23. Cochrane’s Q supports heterogeneity among studies (Q = 72.5 with 2 degrees of freedom, *p* < 0.0001), as did I^2, estimated as 97% (95%, 99%). Tau^2 was estimated as 0.16 (0.29, 47.3), suggesting a low variance of the estimated treatment effect size.

The psychostimulant meta-analysis of 14 studies [87,88,89,90,91,92,93,94,95,96,97,98,99,100] yielded random effects model treatment effects and 95% confidence interval of −0.05 (−0.11, 0.02), *p* = 0.14. Cochrane’s Q did not support heterogeneity among studies (Q = 11.99 with 13 degrees of freedom, *p* = 0.53), and nor did I^2, estimated as 0.0% (0.0%, 55%). Tau^2 was estimated as 0 (0.0, 0.03), suggesting a low variance of the estimated treatment effect size.

The SSRI/antidepressant meta-analysis of 2 studies [101,102], one of which had a significant negative-trending effect size, while the other was slightly positive-trending, yielded random effects model treatment effects of −0.25 (−0.88, 0.38), *p* = 0.44. Cochrane’s Q supports heterogeneity among studies (Q = 6.84 with 1 degrees of freedom, *p* = 0.009), as did I^2, estimated as 85% (41%, 96%). Tau^2 was estimated as 0.18, suggesting a low variance of the estimated treatment effect size.

The steroid meta-analysis of 2 studies [103,104], both of which had significant negative-trending effect sizes, yielded the random effects model treatment effects of −0.94 (−1.21, −0.67), *p* <0.0001. Cochrane’s Q does not support heterogeneity among studies (Q = 0.06 with 1 degrees of freedom, *p* = 0.80), nor did I^2, estimated as 0%. Tau^2 was estimated as 0.0, suggesting a low variance of the estimated treatment effect size.

## 6. Discussion

In our study, we found that the overall meta-analysis of all CRF treatment studies showed significant reduction of fatigue with treatment effect of −0.29. Our meta-analysis suggests significant reduction of CRF with Corticosteroids. Metanalysis of psychostimulants (Methylphenidate, Modafinil, Armodafinil), Ginseng, Guarana, Megestrol, Mistletoe, and antidepressants did not show significant reduction in CRF. Further studies are needed.

As compared to recent meta-analysis of pharmacological treatment for CRF studies (last 6 years) [62,63,105,106,107,108,109,110], the results of our study suggests significant reduction of CRF with Corticosteroids, whereas no significant reduction in CRF with psychostimulants such as Methylphenidate, Modafinil, Armodafinil, Ginseng, Guarana, Megestrol, Mistletoe, and SSRI/antidepressants. Our study was unique as it included only RCT’s with a control or placebo, publications in English, and included both nutraceuticals and pharmaceuticals used to treat CRF as most often patients prefer to use both the nutraceuticals and pharmaceuticals if they decide to address their CRF with medications [32]. In addition, in this study we employed more rigorous methods including strict eligibility criteria, use of Physiotherapy Evidence Database (PEDro) scale to assess the quality of the included RCT studies, and Cochrane risk assessment tool to assess risk bias of the RCT’s. Some of the published metanalysis articles focused on specific stage of disease or type of medication. Roji et al. [106], and Junior et al. [107], focused on placebo. Qu et al. [108], Minton et al. [109], and Gong et al. [110], focused on psychostimulants drugs. Mucke et al. [62] focused mainly on patients receiving palliative care. Our study is unique in contrast to other recent published metanalysis using pharmaceuticals and nutraceuticals in that we included all stages and types of cancer patient populations (early, advanced, cancer survivors as well as cancer types) as well as pharmaceuticals and nutraceuticals. Additionally, we excluded patients using erythropoietic agents as they no longer used to treat CRF due to concerns of increased risk for cardiovascular events and tumor growth [111,112]. Pharmacological or nutraceutical agents (Melatonin, Minocycline) which had only one eligible study were excluded from metanalysis of the agent as the results were more a representative of the single randomized control study rather than a metanalysis of various studies using the agent.

Despite the mixed findings of benefit of various interventions for CRF in our study, it is very early to state any of these interventions are not effective in treatment of CRF due to the limited clinical trials conducted using validated outcomes and in well-defined homogenous cancer patients. Further research is necessary to evaluate which subgroup of cancer patients these interventions will be most likely benefit.

The result from this study regards to the beneficial effects of corticosteroids on CRF is consistent with the results of prior studies using steroids [113,114,115,116] which were excluded as they did not meet all the eligibility criteria of our study but support the use of this agent. However, recent advent of immunotherapy as one of the important cancer treatment agents limits its use of corticosteroids, therefore other agents should be considered among patients on immunotherapy [117].

In contrast to other pharmaceutical and nutraceuticals, psychostimulants specifically Methylphenidate is the most investigated medication for the treatment of CRF. The results of the metanalysis showed non-significant trend towards benefit. These results are interesting as patients in clinical practice often report interest and benefit from the medication. Perhaps a possible reason for mixed results is that we have not targeted the intervention is a specific subgroup of patients (e.g., fatigued cancer patients with anxiety or depression or drowsiness) [90] or we have not used Methylphenidate in combination with other CRF treatments (e.g., exercise) as it may not target all the pathophysiologic mechanisms causing fatigue (especially physical fatigue) [118,119]. In clinical practice in view of the mixed results from metanalysis, as well as side effect profile which includes risk of addiction one should only consider using Methylphenidate in a short term and on a trial basis. In a study by our team [120], we found that patients who showed improvement in CRF (response) after 1 day of treatment of Methylphenidate will most likely have a benefit from methylphenidate treatment for CRF. Therefore, in clinical practice for patients appropriate to use Methylphenidate for CRF, one may consider a trial of Methylphenidate for 1 or 2 days and then extend treatment further on an empirical basis if beneficial with close monitoring.

Various methodological factors can make the interpretation of results of our study challenging: (a) CRF is a subjective symptom and various tools were used in the published clinical trials. (b) Most of the studies used statistical significance rather than clinically relevant improvement as a measure to conclude benefit [121]. Therefore, there were limited details in the published studies which show that improvement in subjective fatigue would result in actual improvement in physical function, activity or interference in daily activity. (c) Finally, during the analysis phase (see Figure 1) we had to exclude several studies due to availability of limited data to conduct the meta-analysis.

Finally, CRF is a complex multidimensional syndrome due to various physical, cognitive, psychosocial factors involving brain, muscle, cognition and effecting various pathophysiological changes including inflammation, neuro-immuno-pituitary adrenal axis, mitochondrial pathways [36]. Using a single agent may not target all the causes of this complex multifactorial syndrome. Hence, future studies targeting various predominant causative mechanisms in specific patient, i.e., multimodal personalized therapy similar to the current management of cancer specific therapy should be considered [122].

## 7. Conclusions

In this study the results of metanalysis of published studies for the treatment of CRF showed significant reduction of cancer related fatigue after treatment with Pharmacological, Nutraceutical and Phytopharmaceutical interventions with a pooled standardized treatment effect of −0.29. Metanalysis of Corticosteroids studies showed significant reduction in CRF. Metanalysis of agents such as the psychostimulants (Methylphenidate, Modafinil, Armodafinil), Ginseng, Guarana, Megestrol, Mistletoe, and antidepressants did not show significant reduction in CRF. Further studies are needed.

## Figures and Tables

**Table 1:** Published Cancer Related Fatigue Pharmacological, Nutraceutical and Phyto Pharmaceuticals for Treatment Trials and Relevant Summaries.

Author, Year	Study Design	Treatment	Fatigue Scale	Patient Number	Population	Treatment Duration	Conclusion	PSS
**Meta-Analysis 1: Ginseng**								
Barton, 2013 [73]	Randomized double-blind placebo-controlled study	Wisconsin ginseng 2000 mg/day	MFSI-SF	364	Cancer patients diagnosed in <=2 years (except brain or CNS lymphoma) undergoing or having undergone curative intent treatment	8 weeks	Wisconsin ginseng improves CRF after 8 weeks	9
Jiang, 2015 [74]	Randomized study	3000 mg of fermented red ginseng extract daily for 60 days	FSI	60	Non-small cell lung cancer patients treated with chemotherapy	60 days	Fermented red ginseng extract significantly improved CRF in patients who received fermented red ginseng.	5
Kim HS, 2017 [75]	Randomized, double-blind, placebo-controlled study	Red ginseng 3000 mg/day	BFI	30	Female patients with ovarian cancer	12 weeks	CRF significantly improved after 12 weeks of treatment with Red ginseng	9
Yennurajalingam, 2017 [76]	Randomized double-blind placebo-controlled Study	Oral panax ginseng extract 800 mg/day	FACIT-F	133	Mixed Cancer patients with cancer-related fatigue	4 weeks	Ginseng and placebo result in significant improvement in cancer related fatigue. Ginseng was not significantly superior to placebo after 4 weeks of treatment.	9
Guglielmo, 2020 [77]	Randomized, double-blind, placebo controlled, phase II trial	1000 mg of American ginseng/day	BFI	32	Head & neck cancer survivors	8 Weeks	American ginseng arm was not significantly different from placebo arm in post treatment Head & neck cancer survivors.	9
Kim, 2020 [78]	Randomized, double blinded, placebo-controlled, parallel phase III trial	2000 mg Korean red ginseng/day	BFI	438	Colorectal cancer patients on mFOLFOX-6 chemotherapy regimen	16 Weeks	Korean Red Ginseng reduced CRF compared with placebo.	9
**Meta-Analysis 2: Guarana**								
Da Costa Miranda V, 2009 [79]	Double-blind placebo controlled, randomized clinical with crossover	Guarana 75 mg daily	BFI	36	Breast cancer patients undergoing adjuvant radiation therapy	2 weeks	CRF was not significantly reduced by guarana over placebo in patients with breast cancer undergoing to radiation therapy	8
De Oliveira Campos, 2011 [80]	Randomized, double-blind, placebo-controlled crossover trial	Guarana 100 mg daily	FACIT-F	75	Breast cancer patients to start the first cycle of systemic chemotherapy	3 weeks	Guarana significantly improved CRF in breast cancer patients receiving systemic chemotherapy.	9
del Giglio, 2013 [81]	Randomized, placebo-controlled study	Guarana extract 75 mg daily	BFI	40	Solid tumors	3 weeks	No significant differences could be seen between the placebo and Guarana arms in the randomized phase	5
**Meta-Analysis 3: Megestrol**								
Bruera, 1998 [82]	Randomized, double-blind, crossover study	Megestrol acetate 480 mg daily	PFS	84	Advanced, solid tumor patients not responsive to hormone therapy	10 days	There was a significant improvement in 2 of the 3 factors measured by the Piper Fatigue Scale and in the overall fatigue score in the Megestrol group.	8
Westman, 1999 [83]	Randomized, double-blind, placebo-controlled study	Megestrol acetate 320 mg daily	EORTC QLQ-C30	255	Advanced, solid tumor patients not responsive to hormone therapy	12 weeks	Megestrol acetate does not appear to improve CRF in Megestrol group compared to placebo.	9
**Meta-Analysis 4: Mistletoe**								
Kim 2012 [84]	Randomized, controlled Trial	Mistle toe extract 20 mg three times a week	EORTC QLQ-C30	32	Gastric cancer (stage Ib or II) who were waiting for oral chemotherapy	24 weeks	No significant difference in fatigue between mistletoe and control group.	6
Tröger, 2009 [85]	Randomized controlled Trial	mistletoe extract 0.01–5 mg three times a week.	EORTC QLQ-C30	95	Breast cancer during six cycles of consecutive treatment with CAF	3 weeks	Symptoms of fatigue decreased in mistletoe group compared to the control group.	6
Tröger, 2014 [86]	Single-center, group-sequential, randomized controlled study	mistletoe extract 0.01–10 mg three times a week.	EORTC QLQ-C30	220	Locally advanced or metastatic pancreatic carcinoma	12 Months	Mistletoe treatment significantly improves the quality of life, including CRF.	6
**Meta-Analysis 5: Psychostimulant**								
Auret KA, 2009 [87]	Randomized, double-blind, placebo-controlled trial	Dexamphetamine 20 mg daily	BFI	50	Patients with advanced cancer receiving palliative care	1 week	Fatigue intensity was not significantly different between the Dexamphetamine and placebo arms.	9
Berenson JR, 2015 [88]	Double-blind, randomized, placebo-controlled, crossover study	Armodafinil 150 mg once daily.	FACIT-F	50	Patients with multiple myeloma	8 weeks	No significant difference between Methylphenidate and Placebo after 4 weeks.	9
Bruera E, 2006 [89]	Randomized, double blinded placebo controlled clinical trial	Methylphenidate 5 mg was given every 2 h, as needed, up to 20 mg per day.	FACIT-F	112	Cancer patients with fatigue	1 week	Methylphenidate was not significantly superior to placebo after 1 week of treatment.	9
Bruera E, 2013 [90]	Randomized, double blinded placebo controlled clinical trial	Methylphenidate 5 mg every 2 h as needed up to 20 mg per day	FACIT-F	190	Patients with advanced cancer	2 weeks	Methylphenidate and a nursing telephone intervention alone or combined were not superior to placebo in improving CRF.	9
Butler JM, 2007 [91]	Randomized placebo controlled double blind clinical trial	Methylphenidate 5 mg twice daily, starting by day 5 of radiation treatment, escalated by 5 mg twice daily to a maximum of 15 mg twice daily for 8 weeks	FACIT-F	68	Metastatic or histologic confirmed primary brain tumor receiving radiation therapy	8 weeks	Prophylactic use of d-Methylphenidate in brain tumor patients undergoing radiation therapy did not result in an improvement in CRF.	8
Centeno, 2020 [92]	Randomized double-blind placebo- controlled clinical trial	Methylphenidate 10–25 mg/day	FACIT-F	77	Patients with advanced cancer	6 Days	Methylphenidate was not significantly better than placebo to treat cancer- related fatigue.	9
Fan Mar, G.H, 2008 [93]	Randomized double-blind placebo controlled clinical trial	d-Methylphenidate 5 mg twice a day, then increased 1 week later to 10 mg bid until the end of the final cycle of chemotherapy.	FACIT-F	57	Women undergoing adjuvant chemotherapy for breast cancer	End of chemotherapy	There are no trends to suggest that d-Methylphenidate, taken concurrently with adjuvant chemotherapy, improves CRF or quality of life.	8
Hovey E, 2014 [94]	Randomized, double blinded placebo controlled clinical trial	Modafinil 100 mg twice daily	MDASI	88	Patients with metastatic prostate or breast cancer undergoing docetaxel chemotherapy	2 weeks	Modafinil treatment did not significantly improve CRF compared to placebo.	9
Jean-Pierre P, 2010 [95]	Randomized, double blinded placebo controlled clinical trial	Modafinil 100 mg started on day 5 or day 10 of the second cycle of chemotherapy for 3 days and then increased to the full dose of 200 mg and continued on this regimen until day 7 of study cycle 4	MDASI	877	Mixed cancer types who were beginning a cancer-treatment course of 4 planned cycles of chemotherapy	Day 7 of study chemotherapy cycle 4	No significant differences in the control of cancer-related fatigue between modafinil and placebo.	8
Lee EQ, 2016 [96]	Randomized, double blinded placebo controlled clinical trial	Armodafinil 150 mg daily	FACIT-F	81	Patients with grade 2–4 glioma scheduled to receive radiotherapy	Day 42	No significant differences were found between armodafinil and placebo arm in CRF improvement.	9
Lower EE, 2009 [97]	Randomized double blind, placebo-controlled, parallel group study	d-Methylphenidate 10 mg a day increasing to a maximum of 50 mg per day over 8 weeks	FACIT-F	154	Patients with cancer (excluding primary or metastatic brain cancer)	Week 8	Compared with placebo, d-Methylphenidate treated subjects demonstrated a significant improvement in CRF	9
Moraska AR, 2010 [98]	Randomized, double blinded placebo controlled clinical trial	Long-acting methylphenidate 18 mg tablet; one tablet on days 1 through 7, two tablets on days 8 through 14, and three tablets on days 15 through 28.	BFI	148	Mixed tumor type Cancer patients	4 weeks	Long-acting methylphenidate did not significantly decrease CRF compared to placebo.	9
Page BR, 2015 [99]	Double-blind placebo-controlled randomized clinical trial	Armodafinil 150 mg daily	FACIT-F	54	Patients with primary brain tumor (malignant or benign/low grade) receiving either partial or WBRT	4 weeks	There were no significant differences in CRF severity between armodafinil and placebo at the end-radiation therapy or 4-week post-radiation therapy.	9
Spathis A, 2014 [100]	Double-blind, placebo-controlled, randomized clinical trial	Modafinil 100 mg on days 1 to 14 and then 200 mg on days 15 to 28.	FACIT-F	208	Adults with advanced non-small cell lung cancer or recurrent disease after surgery or radiotherapy	4 weeks	There was no difference of CRF between Modafinil and Placebo arms.	9
**Meta-Analysis 6: SSRI/Antidepressant**								
Morrow, 2003 [101]	Double-blind, placebo-controlled, randomized clinical trial	paroxetine 20 mg daily	MAF	549	Patients with solid cancer scheduled to begin chemotherapy	8 Weeks	Paroxetine did not result in improvement of CRF in patients with solid cancer receiving chemotherapy.	9
Ashrafi F. 2018 [102]	Randomized, double-blind, placebo-controlled trial	bupropion SR tablet 150 mg daily	FACIT-F	57	Both solid and non-solid cancer patients	4 weeks	Significant improvement in CRF and quality of life in the bupropion compared to placebo arm.	9
**Meta-Analysis 7: Steroids**								
Paulsen, 2014 [103]	Randomized, placebo-controlled, double-blind trial	Methylprednisolone 32 mg daily	EORTC QLQ-C30	50	Advanced cancer patients	1 week	Methylprednisolone 32 mg daily improved fatigue, appetite loss, and patient satisfaction.	10
Yennurajalingam, 2013 [104]	Double-blind, randomized, placebo-controlled trial	Dexamethasone 8 mg daily	FACIT-F	132	Advanced cancer patients	2 weeks	Dexamethasone is more effective than placebo in CRF and quality of life in patients with advanced cancer.	9

**Table 2:** Risk Bias for Studies Included in the Meta-Analysis.

Author, Year	Random Sequence Generation (Selection Bias)	Allocation Concealment (Selection Bias)	Blinding of Participants and Personnel (Performance Bias)	Blinding of Outcome Assessment (Detection Bias)	Incomplete Outcome Data (Attrition Bias)	Selective Reporting (Reporting Bias)	Other Sources of Bias
**Meta-Analysis 1: Ginseng**							
Barton, 2013 [73]	Low	Low	Low	Low	Moderate	Low	NI
Jiang, 2015 [74]	Low	High	High	High	Low	Low	NI
Kim HS, 2017 [75]	Low	Moderate	Low	Low	Low	Low	NI
Yennurajalingam, 2017 [76]	Low	Low	Low	Low	Low	Low	NI
Guglielmo, 2020 [77]	Low	Moderate	Low	Moderate	Moderate	Moderate	Low
Kim, 2020 [78]	Low	Low	Low	Moderate	Moderate	Moderate	Low
**Meta-Analysis 2: Guarana**							
Da Costa Miranda V, 2009 [79]	Low	High	Low	Low	Low	Low	NI
De Oliveira Campos, 2011 [80]	Low	Moderate	Low	Low	Moderate	Low	NI
del Giglio, 2013 [81]	Low	High	High	High	Low	Low	NI
**Meta-Analysis 3: Megestrol**							
Bruera, 1998 [82]	Low	Low	Low	Low	Moderate	Low	NI
Westman, 1999 [83]	Low	Low	Low	Low	Moderate	Low	NI
**Meta-Analysis 4: Mistletoe**							
Kim 2012 [84]	Low	Low	High	High	Low	Low	NI
Tröger, 2009 [85]	Low	High	High	High	Low	Low	NI
Tröger, 2014 [86]	Low	High	High	High	Low	Low	NI
**Meta-Analysis 5: Psychostimulant**							
Auret KA, 2009 [87]	Low	Low	Low	Low	Low	Low	NI
Berenson JR, 2015 [88]	Low	Low	Low	Low	Low	Low	NI
Bruera E, 2006 [89]	Low	Low	Low	Low	Low	Low	NI
Bruera E, 2013 [90]	Low	Low	Unclear *	Low	Low	Low	NI
Butler JM, 2007 [91]	Unclear *	Low	Low	Low	Moderate	Low	NI
Centeno, 2020 [92]	Low	Low	Low	Low	Low	Moderate	NI
Fan Mar, G.H, 2008 [93]	Low	Low	Low	Low	Low	Low	NI
Hovey E, 2014 [94]	Low	Low	Low	Low	Low	Low	NI
Jean-Pierre P, 2010 [95]	Low	Low	Low	Low	Low	Low	NI
Lee EQ, 2016 [96]	Low	Low	Low	Low	Low	Low	NI
Lower EE, 2009 [97]	Low	Low	Low	Low	Low	Low	NI
Moraska AR, 2010 [98]	Low	Low	Low	Low	Low	Low	NI
Page BR, 2015 [99]	Low	Low	Low	Low	Low	Low	NI
Spathis A, 2014 [100]	Low	Low	Low	Low	Low	Low	NI
**Meta-Analysis 6: SSRI/Antidepressant**							
Morrow, 2003 [101]	Low	Low	Low	Low	Low	Low	NI
Ashrafi F. 2018 [102]	Low	Low	Low	Low	Low	Low	NI
**Meta-Analysis 7: Steroids**							
Paulsen, 2014 [103]	Low	Low	Low	Low	Low	Low	NI
Yennurajalingam, 2013 [104]	Low	Low	Low	Low	Low	Low	NI

*: There is insufficient information reported to make a judgement on risk of bias. NI, No information.

## Data Availability

The data presented in this study are available on request from the corresponding author. The data are not publicly available due to privacy.

## Abbreviations

**BFI**, Brief Fatigue Inventory. **EORTC QLQ-C30**, European Organization for the Research and Treatment of Cancer Quality of Life Questionnaire. **FACIT-F**, Functional Assessment of Chronic Illness Therapy–Fatigue. **FSI**, Fatigue Symptom Inventory. **MAF**, Multidimensional Assessment of Fatigue. **MDASI**, The MD Anderson Symptom Inventory. **MFSI-SF**, Multidimensional Fatigue Symptom Inventory. **PFS**, Piper Fatigue Scale. Note: All studies in Table 1 had placebo or other interventions or standard care as comparison arms. PSS- Physiotherapy Evidence Database (PEDro) scoring systems
